# Imaging of non-neoplastic duodenal diseases. A pictorial review with emphasis on MDCT

**DOI:** 10.1007/s13244-018-0593-6

**Published:** 2018-01-31

**Authors:** Sergi Juanpere, Laia Valls, Isabel Serra, Margarita Osorio, Arantxa Gelabert, Albert Maroto, Salvador Pedraza

**Affiliations:** 1 Department of Diagnostic Imaging Institute (IDI) and Girona Biomedical Research Institute (IDIBGI), Dr. Josep Trueta University Hospital, Av/ Francia s/n, Girona, Spain; 2 Department of Gastroenterology, Dr. Josep Trueta University Hospital, Girona, Spain

**Keywords:** Duodenum, MDCT, MRI, Duodenal lesions, Imaging

## Abstract

**Abstract:**

A wide spectrum of abnormalities can affect the duodenum, ranging from congenital anomalies to traumatic and inflammatory entities. The location of the duodenum and its close relationship with other organs make it easy to miss or misinterpret duodenal abnormalities on cross-sectional imaging. Endoscopy has largely supplanted fluoroscopy for the assessment of the duodenal lumen. Cross-sectional imaging modalities, especially multidetector computed tomography (MDCT) and magnetic resonance imaging (MRI), enable comprehensive assessment of the duodenum and surrounding viscera. Although overlapping imaging findings can make it difficult to differentiate between some lesions, characteristic features may suggest a specific diagnosis in some cases. Familiarity with pathologic conditions that can affect the duodenum and with the optimal MDCT and MRI techniques for studying them can help ensure diagnostic accuracy in duodenal diseases. The goal of this pictorial review is to illustrate the most common non-malignant duodenal processes. Special emphasis is placed on MDCT features and their endoscopic correlation as well as on avoiding the most common pitfalls in the evaluation of the duodenum.

**Teaching points:**

*• Cross-sectional imaging modalities enable comprehensive assessment of duodenum diseases.*

*• Causes of duodenal obstruction include intraluminal masses, inflammation and hematomas.*

*• Distinguishing between tumour and groove pancreatitis can be challenging by cross-sectional imaging.*

*• Infectious diseases of the duodenum are difficult to diagnose, as the findings are not specific.*

*• The most common cause of nonvariceal upper gastrointestinal bleeding is peptic ulcer disease.*

## Anatomy

The duodenum measures about 25 cm to 30 cm in length [1]. It does not form part of the mesenteric small bowel and it has both an extra- and intraperitoneal location [2]. The first portion (*duodenal bulb*) is suspended intraperitoneally and extends from the gastric pylorus to the gallbladder neck. The second portion (*descending duodenum*) extends between the superior and inferior duodenal flexures in the anterior pararenal space. The anatomic space between the duodenum and pancreatic head is the pancreaticoduodenal groove. The major and minor duodenal papillae are usually located in the second part of the duodenum. The third portion (*horizontal duodenum*) runs behind the peritoneum from right to left, crossing over the inferior vena cava and aorta. The major duodenal papilla is located in the third portion in up to 25% of cases [3]; however, other series report a much lower percentage (1.4%) [4]. The fourth portion (*ascending duodenum*) courses superiorly to the duodenojejunal flexure. The duodenum is supplied by the pancreaticoduodenal arcades. The superior pancreaticoduodenal artery is an anatomic landmark between the descending duodenum and the pancreatic head, and the inferior pancreaticoduodenal artery is an anatomic landmark between the horizontal duodenum and uncinate process of the pancreas.

## Imaging modalities

For years, barium studies were the reference standard for depicting and characterizing duodenal structures. However, this approach has been supplanted by fiberoptic endoscopy and cross-sectional imaging techniques, of which, multidetector computed tomography (MDCT) and magnetic resonance imaging (MRI) are the least invasive and most widely available. Both MDCT and MRI have proven very useful in evaluating the intraluminal content, duodenal wall, and extraduodenal space [2]. MDCT has become the modality of choice for evaluating duodenal and periduodenal abnormalities. Neutral contrast agents (e.g., water, polyethylene glycol, or methylcellulose mixed in water) do not cause streaking artefacts and have, thus, proven more effective than traditional positive contrast agents in showing contrast-enhancing duodenal lesions. Nevertheless, iodinated intravenous contrast material is routinely administered in the absence of contraindications. Multiplanar reconstructions and three-dimensional volume-rendered imaging have been shown to help with the confusing anatomy. However, MDCT remains limited for differentiating duodenal wall layers and intramural conditions, which is better done using endoscopic ultrasound. MRI small-bowel follow-through has the advantages of multiplanar imaging capabilities with excellent spatial and contrast resolution, without the need for ionizing radiation or potentially nephrotoxic contrast medium, thus, making it especially attractive for sequential imaging over prolonged periods of time. Regardless of the technique used, suboptimal duodenal distension can obscure discrete abnormalities.

## Congenital anomalies

**Fig. 1:**
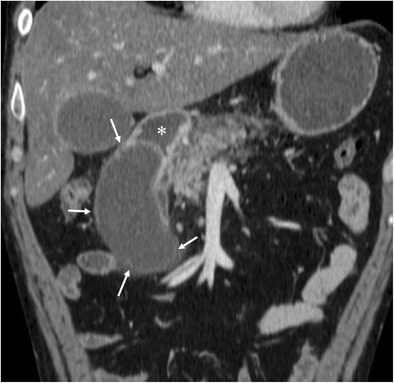
Duodenal duplication cyst. Reformatted coronal contrast-enhanced CT image shows a large well-circumscribed cystic mass (arrows) adjacent to the second duodenal portion. There is no communication with the duodenal lumen (*)

**Fig. 2:**
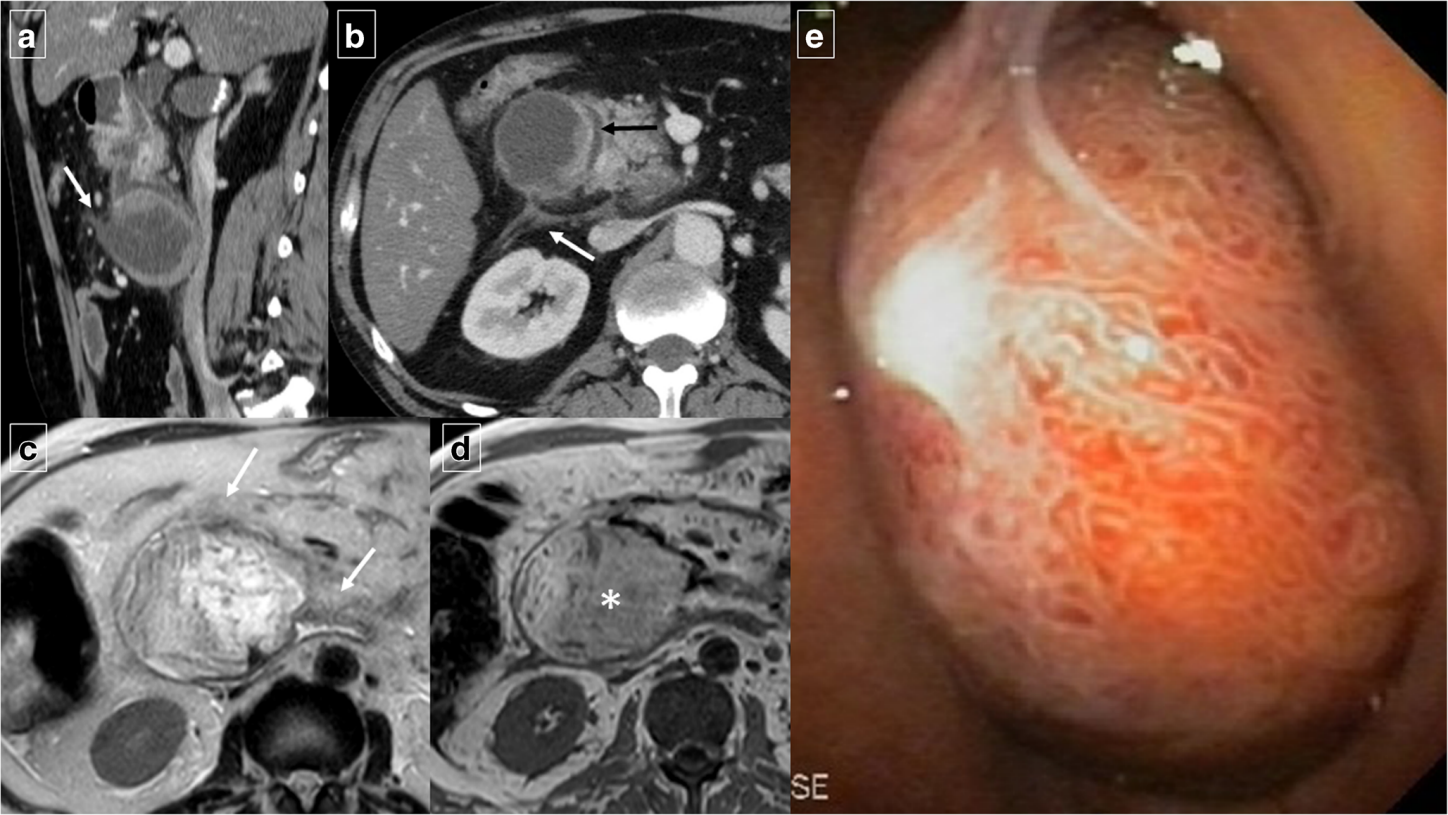
Ruptured duodenal duplication cyst in a 58-year-old man who sustained blunt trauma in a fall down stairs. **a** Reformatted sagittal contrast-enhanced CT shows circumferential wall thickening of the cyst and a disruption in its anterior surface (arrow). **b** Axial CT shows retroperitoneal fluid in the right anterior pararenal space (white arrow). The duodenal lumen is narrowed and displaced medially due to inflammatory changes (black arrow). **c** Axial T2-weighted MRI shows stranding of the periduodenal fat on (arrows). **d** Axial T1-weighted MRI image shows a hyperintensity of the content of the lesion (*). The high signal intensity (more than muscle tissue), in a traumatic context, suggests an haemorrhagic transformation of the cyst. **e** Detail of the fiberoptic endoscopic study

Duplication cysts are infrequent in the gastrointestinal tract, and approximately 12% involve the gastroduodenal region [1, 3]. Duodenal duplication arises most often in the medial wall of the second and third portions of the duodenum. On CT, duodenal duplication cysts typically appear as well-circumscribed, non-enhancing cystic masses with fluid attenuation (Fig. 1), which are hyperintense on T2-weighted MRI images. These duplications do not normally communicate with the duodenal lumen. Although symptoms related to obstruction or superinfection may be present, duodenal duplication cysts are usually asymptomatic and are often discovered incidentally at abdominal imaging. The differential diagnosis includes pancreatic pseudocysts, mesenteric cysts, and choledochal cysts. Rarely, abdominal trauma can cause a duplication cyst to rupture, resulting in fluid leaking out of the system and leading to perilesional or periduodenal fluid collections and wall thickening (Fig. 2). Sometimes, the disruption in the cyst wall can communicate with the duodenal lumen. More rarely, carcinoma can arise inside a duplication cyst [1] and the presence of vegetation, mural polyps or enhanced nodules should raise concern.

**Fig. 3:**
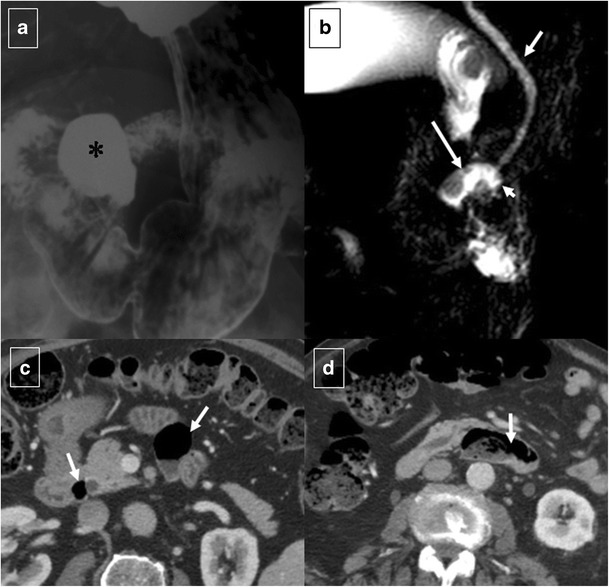
Duodenal diverticula in different patients. **a** Anteroposterior image from a single-contrast upper gastrointestinal series shows a typical outpouching (*) arising from descending duodenum. **b** Magnetic resonance cholangiopancreatography shows a heterogeneous outpouching (large arrow) in the papillary region. Note common bile duct (arrow) and proximal Wirsung duct (arrowhead). **c** Axial CT demonstrates two duodenal diverticula (arrows) in the periampullary region (air-filled) and fourth portion of the duodenum (with air-fluid level). **d** Axial CT reveals a diverticulum (arrow) that contains debris in the third portion of the duodenum. Air bubbles inside the lesion are virtually pathognomonic for a duodenal diverticulum

**Fig. 4:**
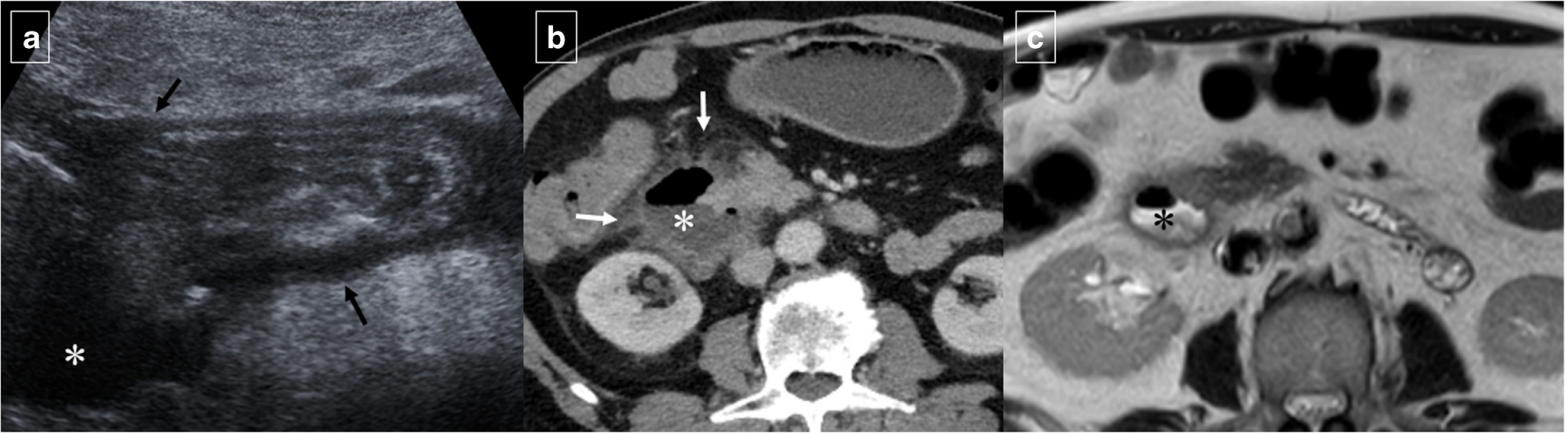
A 67-year-old man with epigastralgia, fever, and food intolerance. **a** Initial abdominal ultrasonography shows diffuse thickening of the different layers of the duodenal wall with a slight amount of free periduodenal fluid (arrows) and an adjacent hypoechoic fluid collection (*). **b** Axial contrast-enhanced CT confirms the presence of a paraduodenal collection with an air-fluid level (*) and surrounding soft-tissue stranding (arrows) caused by acute duodenal diverticulitis. **c** Axial T2-weighted MRI after conservative therapy shows the outpouching from the duodenal wall is smaller and more well-defined; it now has the appearance of a duodenal diverticulum, and the amount of soft-tissue stranding has decreased

**Fig. 5:**
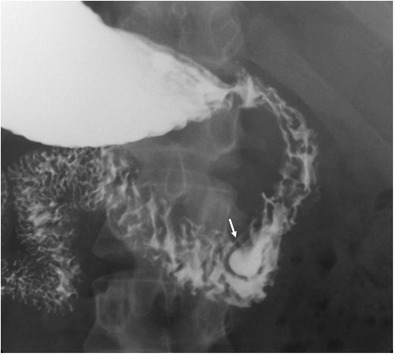
Intraduodenal diverticulum in a 58-year-old woman. Single-contrast upper gastrointestinal study in the right anterior oblique projection shows a well-defined oval lesion (arrow) surrounded by a radiotransparent halo (“windsock” sign). The lesion contains oral contrast material and projects into the true duodenal lumen

Duodenal diverticula are saccular dilations that appear when the mucous and serous layers herniate through a focal mural defect along the pathway of penetrating blood vessels or ducts, most frequently along the medial wall of the second or third segments. The acquired type is more common than the congenital type [2]. They are usually asymptomatic; less than 10% cause symptoms, and only 1% or less require treatment for perforation, haemorrhage, obstruction, or acute diverticulitis [5]. On barium studies, diverticula manifest as contrast-filled outpouchings of the duodenal lumen. On CT, they are seen as a saccular outpouching, which may resemble a mass with air, fluid, an air–fluid level, contrast material, or debris inside (Fig. 3). When filled with fluid, periampullary diverticula may mimic a pancreatic or choledochal cyst. Direct luminal continuity with the duodenum is an important feature to confirm the diagnosis of diverticula. Abdominal imaging of acute duodenal diverticulitis include duodenal wall thickening, stranding of the surrounding soft tissues and adjacent mesenteric or retroperitoneal fat, or surrounding extraperitoneal free air (Fig. 4). Uncommon varieties of duodenal diverticula include acquired traction diverticula as a result of periduodenal inflammatory fibrosis, and true intraluminal diverticula, which consist of a congenital intraluminal membrane with a small aperture that can lead to partial or complete duodenal obstruction [3]. Intraluminal diverticula are seen on barium studies with the classical “wind sock” sign (Fig. 5). Findings similar to those observed on upper gastrointestinal series can be seen on CT: positive oral contrast material is required within the lumen to define the diverticulum wall as a low-density “flap”. However, a collapsed diverticulum on CT can mimic an intraluminal mass/polyp or appear as a subtle thickened wall-like finding. Although an uncommon situation, the differential diagnosis of a contrast-filled diverticulum includes a duodenal intussusception.

**Fig. 6:**
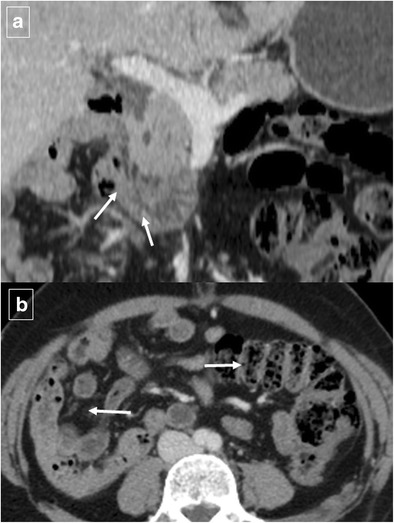
Gastrointestinal nonrotation in a 46-year-old woman. **a** Reformatted coronal contrast-enhanced CT image shows that the third portion of the duodenum (arrows) does not cross the midline of the abdomen. **b** The small bowel is located in the right side of the abdomen and the colon in the left side (arrows) on axial CT image

Malrotation refers to an abnormal position of the intestine within the peritoneal cavity due to the failure of the bowel to complete its rotational sweep during embryological development. Patients with malrotation are predisposed to midgut volvulus, which usually occurs in infancy. The mainstay of imaging in suspected intestinal malrotation is a fluoroscopic upper gastrointestinal series, which rules out malrotation if the duodenojejunal junction is seen to the left of the spine and above the level of the gastric pylorus. On CT, malrotation is diagnosed when the duodenum is not visible between the aorta and the superior mesenteric artery and the superior mesenteric artery (SMA) is located to the right of the superior mesenteric vein. Nonrotation, a subtype of malrotation that is less prone to torsion, is seen as a predominantly right-sided position of the small bowel and predominantly left-sided position of the colon (Fig. 6). Because it is asymptomatic, it is usually discovered incidentally in adults.

**Fig. 7:**
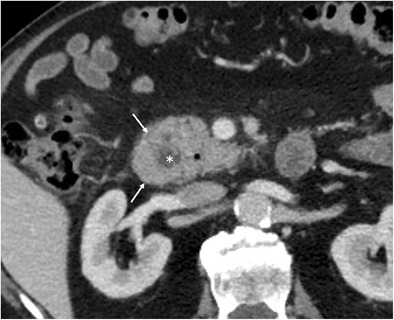
Annular pancreas in a 43-year-old man with a long history of abdominal pain. Contrast-enhanced CT shows the pancreas (arrows) completely encircling the descending duodenum (*)

Annular pancreas is a rare congenital anomaly in which a portion of the duodenum is surrounded by a partial or complete ring of normal pancreatic tissue connected to the head of the pancreas due to incomplete rotation of the ventral pancreatic anlage during embryological development. The second portion of the duodenum is involved in 85% of cases, and the first or third portions in the rest [6]. Up to 75% of patients have other congenital anomalies (tracheoesophageal fistula, imperforate anus, or Hirschsprung’s disease). Annular pancreas normally presents during childhood [6], usually manifesting with symptoms resulting from gastricoutlet obstruction; however, in adults it is mostly asymptomatic, being discovered incidentally in patients with peptic ulcers, duodenal obstruction, or pancreatitis. Clinical findings of gastroduodenal obstruction are found in 21% of patients [7]. A complete ring of pancreatic tissue surrounding the second part of the duodenum diagnoses a complete annular pancreas (Fig. 7), whereas a crocodile jaw appearance of pancreatic tissue anterior and posterior to the duodenum is highly suggestive of incomplete annular pancreas. MRI is superior to CT in showing the annular duct; cholangiopancreatographic MR protocols (MRCP) often will depict an annular duct within the aberrant pancreatic tissue that may communicate with the main pancreatic duct.

## Inflammatory (peri)duodenal processes

**Fig. 8:**
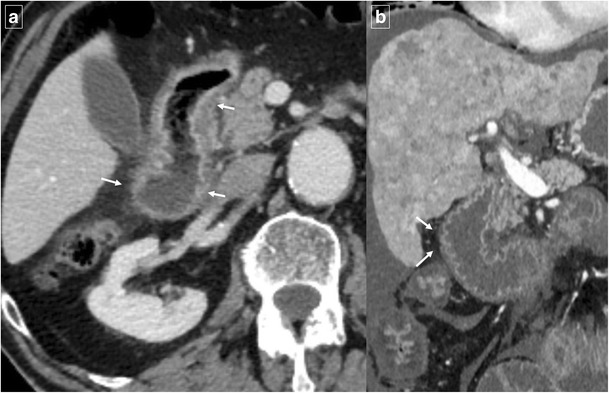
Spectrum of duodenitis in different patients. **a** Axial contrast-enhanced CT shows thickened oedematous duodenal wall with inflammation involving periduodenal fat (arrows) in nonspecific duodenitis. **b** Reformatted coronal contrast-enhanced CT image in a woman with disseminated breast cancer and secondary hepatic insufficiency shows nonspecific mural thickening of the duodenum with mucosal enhancement (arrows)

**Fig. 9:**
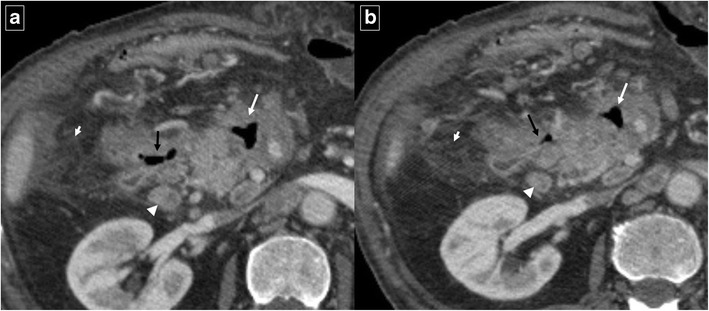
Tuberculosis in a 63-year-old man with constitutional symptoms, fever, and abdominal pain. Axial contrast-enhanced CT shows a masslike appearance (white arrow) that encompasses the superior mesenteric artery and a fistulous pathway that communicates with the duodenal lumen (black arrow). Note the marked extrinsic compression of the third portion of the duodenum. CT also shows periduodenal fat stranding (short arrows) and a necrotic lymph node (arrowhead)

Inflammatory processes in the duodenum are rarely diagnosed at CT. The findings tend to be nonspecific, such as duodenal wall thickening (by submucous edema for example), periduodenal fat stranding, and luminal dilation (Fig. 8). The most common infectious cause of duodenitis is *Helicobacter pylori* [1]. Less common infections include giardiasis, *Escherichia coli O157*, tropical sprue, and norovirus. Infectious processes may induce functional dyspepsia [8]. Duodenal tuberculosis is rare, accounting for only 1% to 2% of all cases of tuberculosis of the gastrointestinal tract [9]. Tuberculosis can affect the duodenum through intrinsic processes or extrinsic compression, or both. Far more common than intrinsic involvement, extrinsic compression results from enlarged peripancreatic or mesenteric root lymph nodes (Fig. 9) and presents with features of duodenal obstruction, whereas those with intrinsic involvement have dyspeptic symptoms. Complications like haemorrhage, perforation, or fistulas are reported in about 5% of cases [9]. Other causes of nonspecific duodenitis include medications (e.g., nonsteroidal anti-inflammatory drugs), radiation therapy, acquired immunodeficiency syndrome, or functional dyspepsia (in up to 40% of cases) [8].

**Fig. 10:**
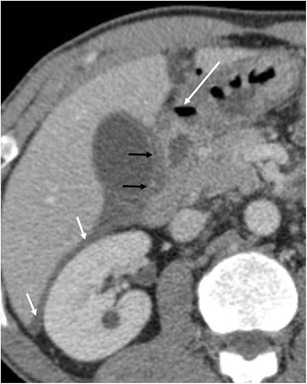
A 48-year-old man who presented with abdominal pain caused by a perforated duodenal ulcer. Axial contrast-enhanced CT shows an irregular air-fluid level (large arrow) adjacent to the duodenal bulb. A leakage of free fluid into the posterior subhepatic space (white arrows) through hepatoduodenal ligament (black arrows) is observed

**Fig. 11:**
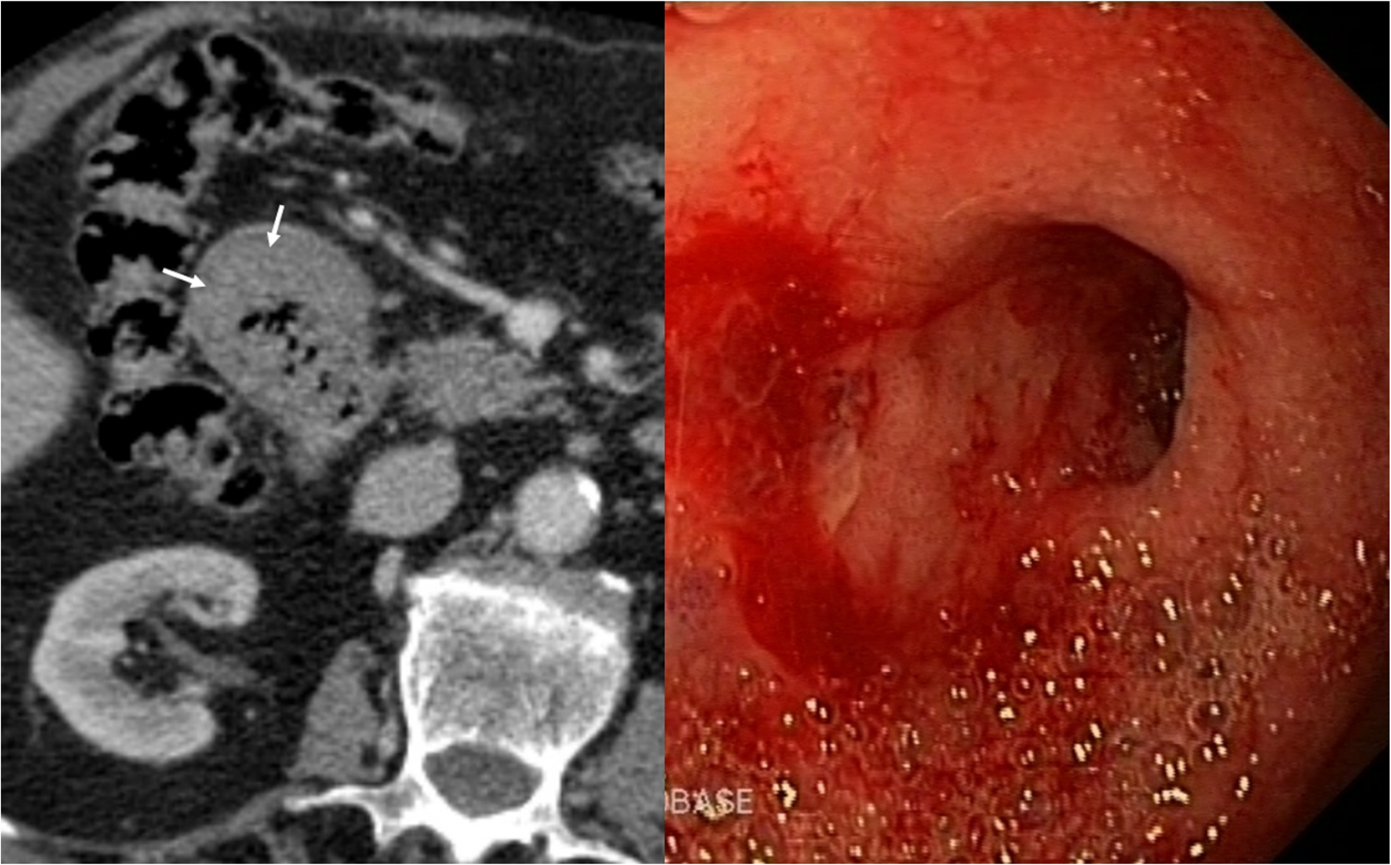
An 88-year-old man who was treated with oral anticoagulants for ischemic heart disease, presented with coffee ground vomitus. Axial contrast-enhanced CT shows homogeneous concentric thickening (arrows) of the duodenal wall at the level of the bulb. Upper endoscopy detected an ulcer in the duodenal bulb with active oozing haemorrhage which was sclerosed with adrenaline

**Fig. 12:**
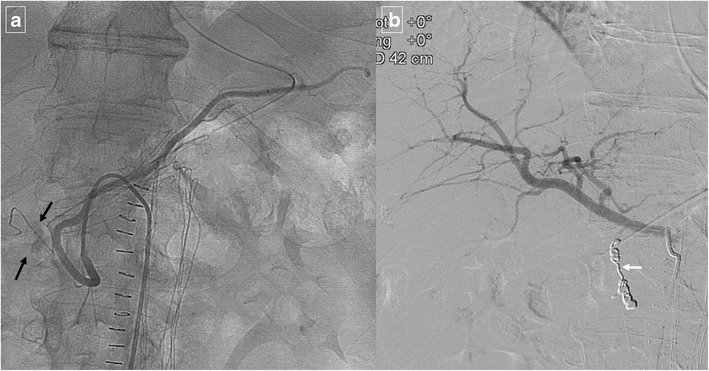
A 63-year-old male smoker with upper digestive bleeding. Initial upper endoscopy showed a large bleeding ulcer on the anterior surface of the duodenal bulb; sclerosing therapy with adrenaline and aethoxysklerol® was unsuccessful. **a** Arteriogram shows contrast medium extravasated from the posterior pancreaticoduodenal arcade (arrows) into the duodenum. **b** Coil embolization of gastroduodenal artery stopped the bleeding

Duodenal peptic ulcers usually occur in the duodenal bulb [1]; more distal ulcers should raise suspicion for an underlying cause, such as Zollinger-Ellison syndrome or Crohn’s disease [3]. Although most commonly detected by upper endoscopy, fluoroscopic upper gastrointestinal series also show most acute duodenal ulcers as a round collection of barium surrounded by a radiolucent halo of edema with associated circumferential or eccentric narrowing of the lumen that can lead to duodenal obstruction. Direct signs of peptic ulcer disease on cross-sectional imaging include focal discontinuity of the mucosal hyperenhancement and identification of luminal outpouching. Another useful indirect sign, which alerts one to possible active inflammation and/or peptic ulcer, is oedematous “stranding” of the periduodenal fat [10]. But cross-sectional imaging is useful for depicting complications of duodenal ulcers, with bleeding the most common complication [10]. Ectopic gas, periduodenal fluid, wall thickening, or contrast material within the periduodenal fat or lesser sac are signs suggesting perforation (Fig. 10). Peptic ulcer disease is the most common cause of nonvariceal upper gastrointestinal bleeding (UGIB) (Fig. 11) [11], and the most common signs of acute bleeding are hematemesis and melena. The mortality associated with peptic ulcer bleeding is 5% to 10% [12]. Early endoscopy aims to determine the cause of bleeding, ascertain prognosis, and administer endoscopic therapy if indicated. Patients with massive bleeding or hemodynamic instability who have failed at least one attempt for endoscopic intervention often benefit from transcatheter arterial embolization. Angiography in the setting of UGIB is positive for extravasation or abnormal mucosal blush in up to 61% of cases (Fig. 12) [11]. Transcatheter intervention for bleeding involves the infusion of vasoconstricting drugs and/or the mechanical occlusion of the feeding vessels. Primary rates of technical success range from 52% to 94% of patients, with recurrent bleeding requiring repeated embolization procedures in approximately 10% [12]. Uncommon complications include bowel ischemia, secondary duodenal stenosis, and gastric, hepatic, or splenic infarction.

**Fig. 13:**
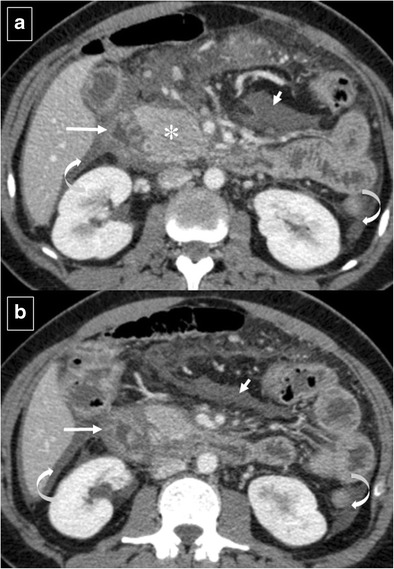
Acute exsudative pancreatitis and duodenal edema in a 50-year-old man. Contrast-enhanced CT shows an enlarged pancreatic head (*) with stranding of the peripancreatic fat and peripancreatic fluid (short arrows). The wall of the duodenum is thickened, and limited mural enhancement is seen secondary to edema (arrows). Also note the pathologic amount of fluid in the upper abdomen (curved arrows)

**Fig. 14:**
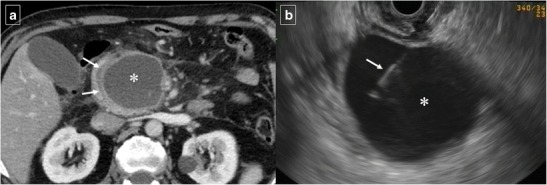
Duodenal compression by pancreatic pseudocyst in a 69-year-old man. **a** Axial contrast-enhanced CT reveals lateral displacement of the descending duodenum (arrows) by a pancreatic fluid collection (*). **b** Detail of an internal drain (arrow) guided by echoendoscopy to improve secondary food intolerance

Pancreatitis is the most common inflammatory process that can affect the duodenum [1, 3]. Severe pancreatitis can lead to duodenal involvement through two mechanisms: the first is direct damage to the duodenal wall by an inflammatory reaction (mural edema or hematoma from disruption of the intramural vasculature) (Fig. 13) [13], and the second is a fibrotic reaction due to compression by pseudocysts or by an enlarged pancreatic head. Pancreatic inflammatory fluid collections and pseudocysts develop in up to 50% of patients with acute pancreatitis [14]. CT shows displacement and extrinsic compression of the descending duodenum without mucosal abnormalities secondary to a well-defined fluid collection with low attenuation (<15 HU) surrounded by a peripheral fibrous capsule (Fig. 14). Higher attenuation values are indicative of a superimposed infection, necrotic tissue, or intracystic haemorrhage.

**Fig. 15:**
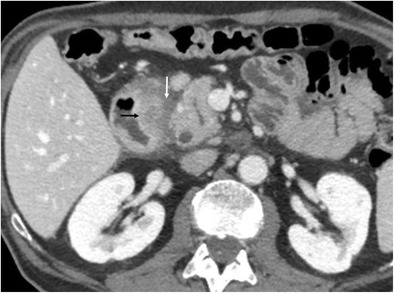
A 48-year-old man with epigastric pain. Contrast-enhanced CT identifies subtle infiltration of soft tissue (white arrow) in the pancreaticoduodenal groove. Duodenal mural thickening secondary to edema is observed on the medial side (black arrow). Note the normal enhancement of the pancreatic head and absence of classic signs of exsudative pancreatitis

**Fig. 16:**
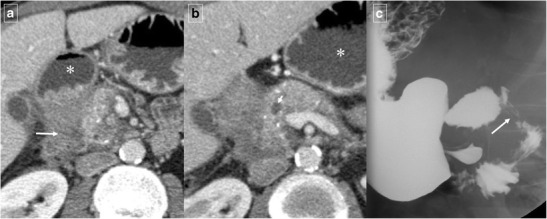
A 55-year-old man with history of alcohol abuse and chronic pancreatitis. Axial contrast-enhanced CT images (**a** and **b**) demonstrate atrophy of the pancreatic parenchyma and ductal dilation (short arrow). A stricture of the duodenal lumen (arrow) caused by fibrotic changes in the pancreaticoduodenal groove is accompanied by gastric dilation (*). **c** Image from a barium study shows a stricture in the proximal duodenum with an abnormal mucosal pattern (arrow)

An uncommon but distinctive form of chronic pancreatitis affecting the pancreatoduodenal space is “groove pancreatitis” [15, 16]. Its pathogenesis includes functional obstruction of the minor papilla, Brunner gland hyperplasia, heterotopic pancreas in the duodenum, and peptic ulcer disease [15, 17]; risk factors include alcohol use or smoking. Groove pancreatitis is most common in men in their fourth and fifth decades of life; it often presents with symptoms of chronic postprandial epigastric pain, nausea, vomiting, and significant weight loss—features that are often more suggestive of an underlying malignancy rather than pancreatitis [17]. Two patterns of groove pancreatitis have been described [16]. In the“pure type” the appearance can range from ill-defined fat stranding and inflammatory changes to frank soft tissue in the groove (Fig. 15). Occasionally, the medial duodenal wall is thickened, and small cysts are often seen within the thickened wall or in the pancreaticoduodenal groove itself. The “segmental type” can be much more difficult to appreciate because involvement of the groove is often obscured by masslike enlargement of the pancreatic head. The diffuse retroperitoneal inflammatory changes seen in acute oedematous pancreatitis are usually absent in groove pancreatitis. Regardless of the type, groove pancreatitis can mimic pancreatic ductal adenocarcinoma, and histologic study might be required to differentiate between them [18]. However, the presence of an abrupt cutoff of the pancreatic duct and the common bile duct and the presence of vascular invasion (including the gastroduodenal artery) are considered the most useful signs in differentiating pancreatic malignancy from groove pancreatitis. In contrast, MRCP can nicely reveal ductal narrowing and irregularity of the distal common bile duct and downstream pancreatic duct in patients with groove pancreatitis. The presence of cysts within the lesion and thickening of the duodenal wall favours groove pancreatitis. Patients with duodenal obstruction in the context of chronic pancreatitis often have dilated pancreatic and biliary ducts (Fig. 16) [7].

**Fig. 17:**
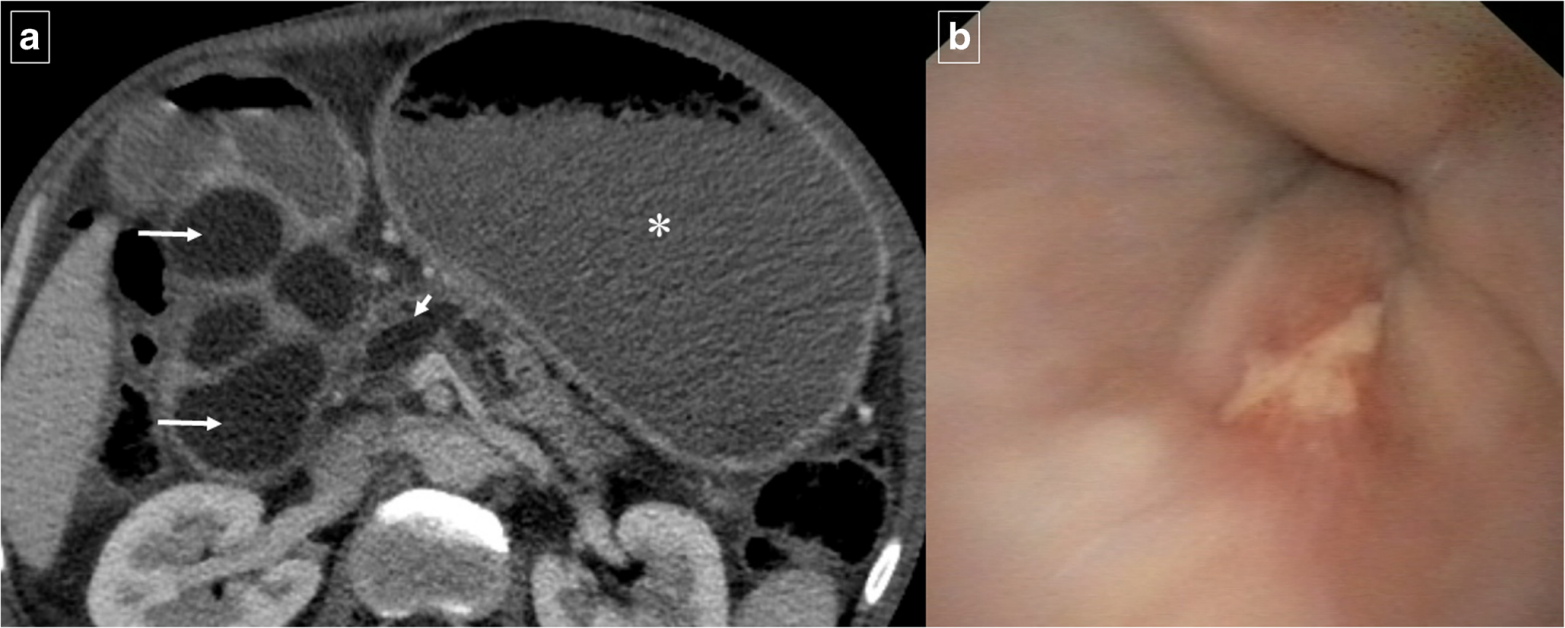
A 56-year-old man with history of alcohol abuse and smoking presented with weight loss. **a** Axial contrast-enhanced CT reveals cystic dystrophy with multiple cystic parietal areas (arrows) in the thickened second duodenal portion causing severe duodenal obstruction with gastric dilation (*). Atrophy of the pancreatic parenchyma and strictures of the pancreatic duct (short arrow) due to chronic pancreatitis. **b** Detail from the upper gastrointestinal endoscopy study showing extrinsic compression of the duodenal lumen

Another group of disorders that shares clinical features with groove pancreatitis is “paraduodenal pancreatitis”, a term which encompasses cystic dystrophy of the heterotopic pancreas, periampullary duodenal wall cyst, pancreatic hamartoma of the duodenal wall, myoadenomatosis, and cystic dystrophy of the duodenal wall [15, 16]. Cystic change is a prominent feature in this group [19] and CT and MRI reveal multiple, elongated or bilobate cysts within the thickened wall of the second portion of the duodenum [20]. Inflammatory changes may result in narrowing of the duodenal lumen with gastric distension (Fig. 17).

### Crohn’s disease

**Fig. 18:**
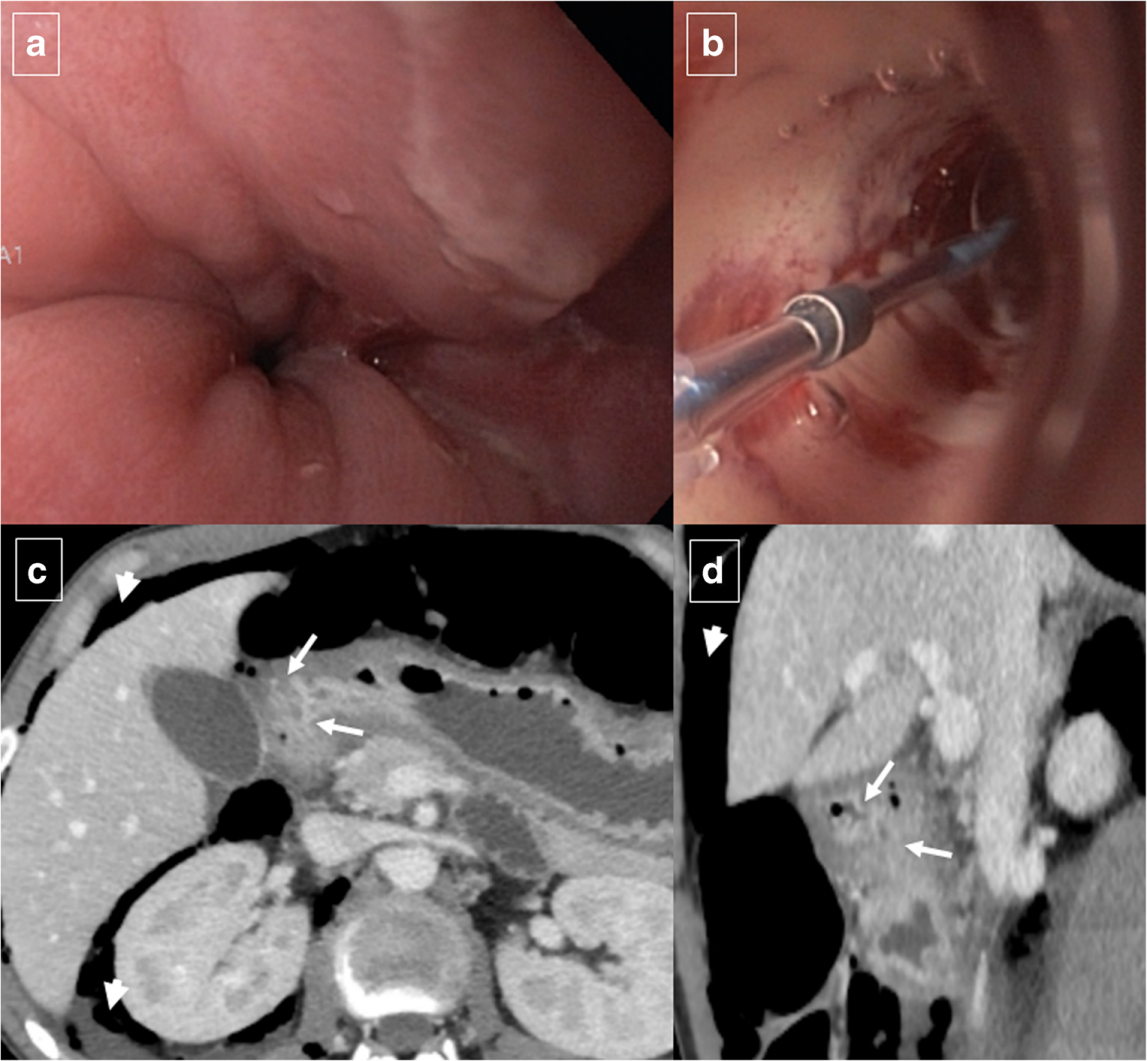
A 24-year-old man with Crohn’s disease who presented with reflux esophagitis, daily vomiting, and food intolerance. **a** Upper endoscopy shows pyloric and bulbar involvement with ulcerated areas and folded hypertrophy that caused stenosis. After the dilation of the stenosis **b** the patient presented acute abdominal pain. Axial **c** and reformatted sagittal **d** contrast-enhanced CT images confirm the suspected perforation (short arrows) and also show the thickened and hyperaemic wall of the duodenum (arrows) with fibrotic bulbar stenosis

Isolated duodenal involvement in Crohn’s disease is rare, being found in roughly 0.5% to 4–5% of cases [1, 3], although the duodenum is affected in 5% to 20% of patients with Crohn’s disease of the small bowel and colon [14]. The purpose of cross-sectional imaging is to determine the number, length, and location of intestinal lesions, to identify areas of stenosis and characterize them as inflammatory or fibrotic, and to identify complications such as fistulas or abscesses. In the stenotic phase, multiple areas of eccentric duodenal stenosis with outward ballooning or sacculation between areas of stricture are often seen (Fig. 18). These characteristics are depicted to greater effect on cross-sectional imaging than on projectional fluoroscopic studies [21].

### Gallbladder pathology

**Fig. 19:**
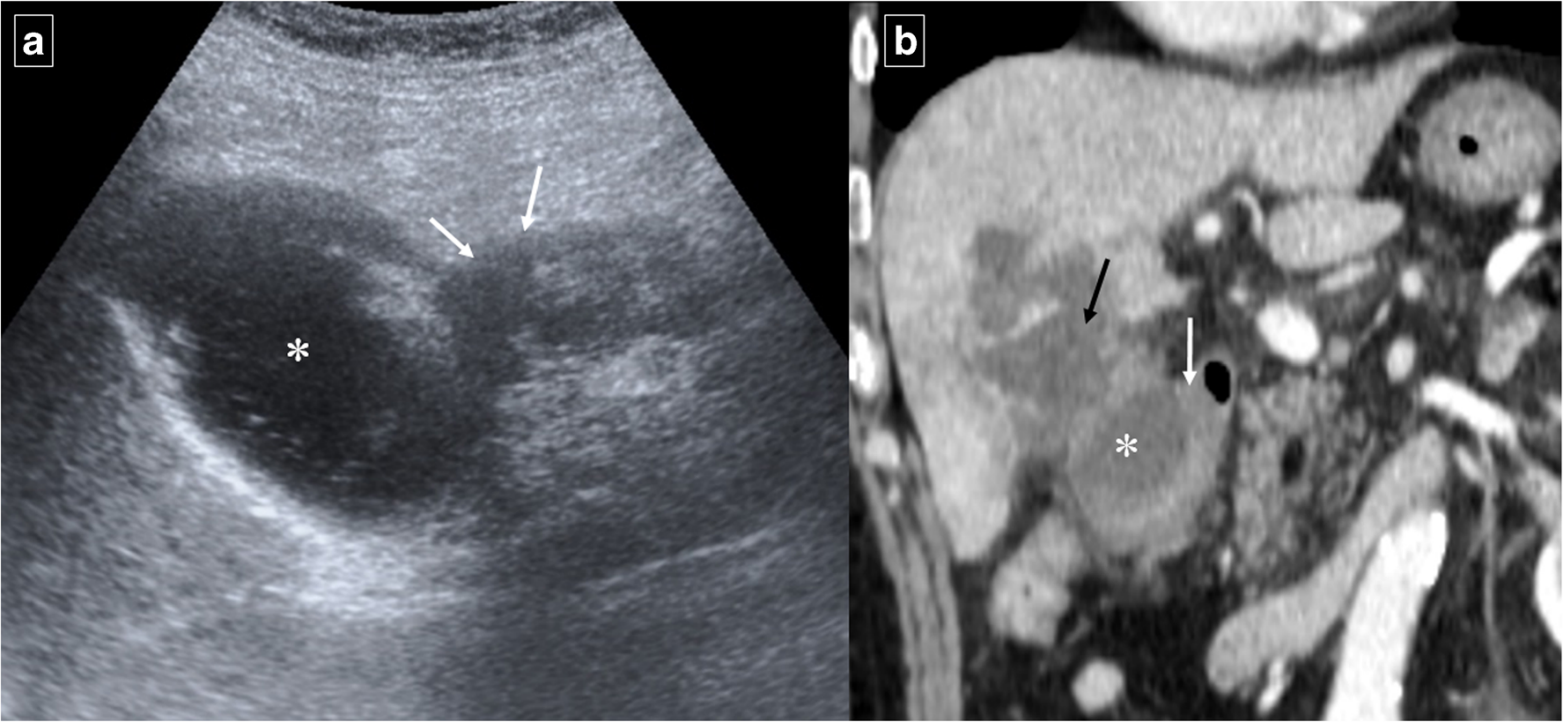
Acute cholecystitis in a 54-year-old man. **a** Ultrasonography identified an enlargement of the lateral wall of the duodenum (white arrows) that was accompanied by a periduodenal fluid collection (*). **b** Reformatted coronal contrast-enhanced CT image shows a distended gallbladder with inaccurate margins (black arrow) and confirms the presence of a periduodenal fluid collection (*). Also note the secondary duodenal wall involvement (white arrow)

**Fig. 20:**
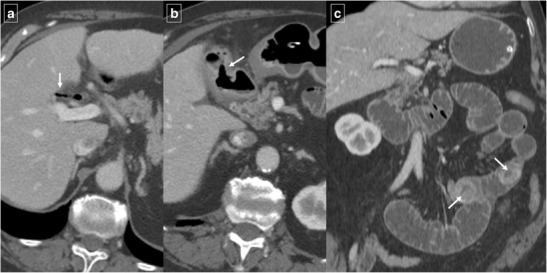
Right upper quadrant pain and gallstone ileus in a 39-year-old woman. Axial contrast-enhanced CT (**a** and **b**) shows air in the bile duct (arrow in **a**), a thick-walled gallbladder, and a fistula to the duodenum (arrow in **b**). Reformatted coronal contrast-enhanced CT image demonstrates two gallstones (arrows) within the proximal ileum with proximal jejunal dilation

Like acute pancreatitis, acute cholecystitis can cause inflammatory thickening of the duodenal wall adjacent to the inflamed gallbladder wall (Fig. 19). If this process is long-standing and severe, a gallstone may erode through the gallbladder wall and into the duodenum, resulting in a “gallstone ileus”. CT findings include pneumobilia, small bowel obstruction, ectopic gallstone, and cholecystoduodenal (or choledocoduodenal) fistulas (Fig. 20). Impaction of a gallstone in the duodenum or stomach via a bilioenteric fistula can cause gastric outlet obstruction (Bouveret syndrome).

## Duodenal trauma

**Fig. 21:**
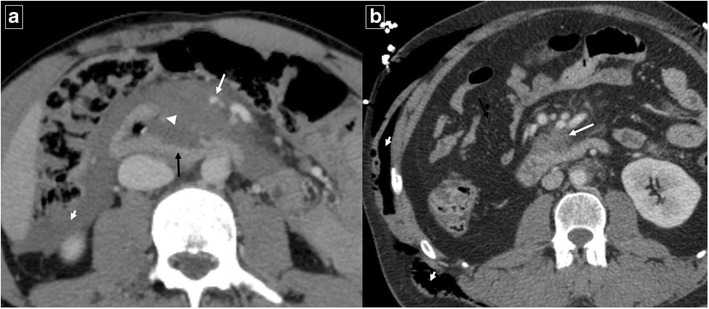
Duodenal trauma in different patients. **a** A 20-year-old man with blunt abdominal trauma. Contrast-enhanced CT shows an extensive pancreaticoduodenal hematoma with displacement of the mesenteric vessels to the left (white arrow) and disruption of the wall of the third duodenal portion (black arrow) with active extravasation of intravenous contrast (arrowhead). Note the abundant retroperitoneal fluid (short arrow). **b** A 45-year-old man involved in a motor vehicle accident. Contrast-enhanced CT shows free retroperitoneal fluid (arrow) adjacent to the third duodenal portion and slight wall thickening or mural edema. Abdominal subcutaneous emphysema (short arrows) secondary to multiple rib fractures and pneumothorax

Traumatic duodenal (and pancreatic) injuries are relatively rare, accounting for less than 2% of all abdominal injuries [3, 22]. Penetrating trauma and blunt abdominal trauma are well-known mechanisms of duodenal injury. When duodenal or pancreatic injuries are present in traumatic patients, and they are not correctly identified as a result of missed finding and/or diagnostic delay, complications can occur in 30% to 60% of the cases [23]. CT is the primary imaging modality for assessing abdominal trauma. Duodenal contusions and hematomas may manifest as a hyperattenuating, thickened duodenal wall; intramural gas; and fluid in the retroperitoneum or stranding of the peripancreatic fat as a result of mural haemorrhage (Fig. 21). Extraluminal gas in the retroperitoneum, discontinuity of the duodenal wall, and extravasation of positive contrast material (if administered) into the retroperitoneum are direct signs of duodenal perforation. Active bleeding must be ruled out first. It is critical to distinguish between a duodenal wall hematoma, which can be treated conservatively, and perforation, which requires surgical intervention [22]. Gastric outlet obstruction may develop later.

## Vascular pathologies

**Fig. 22:**
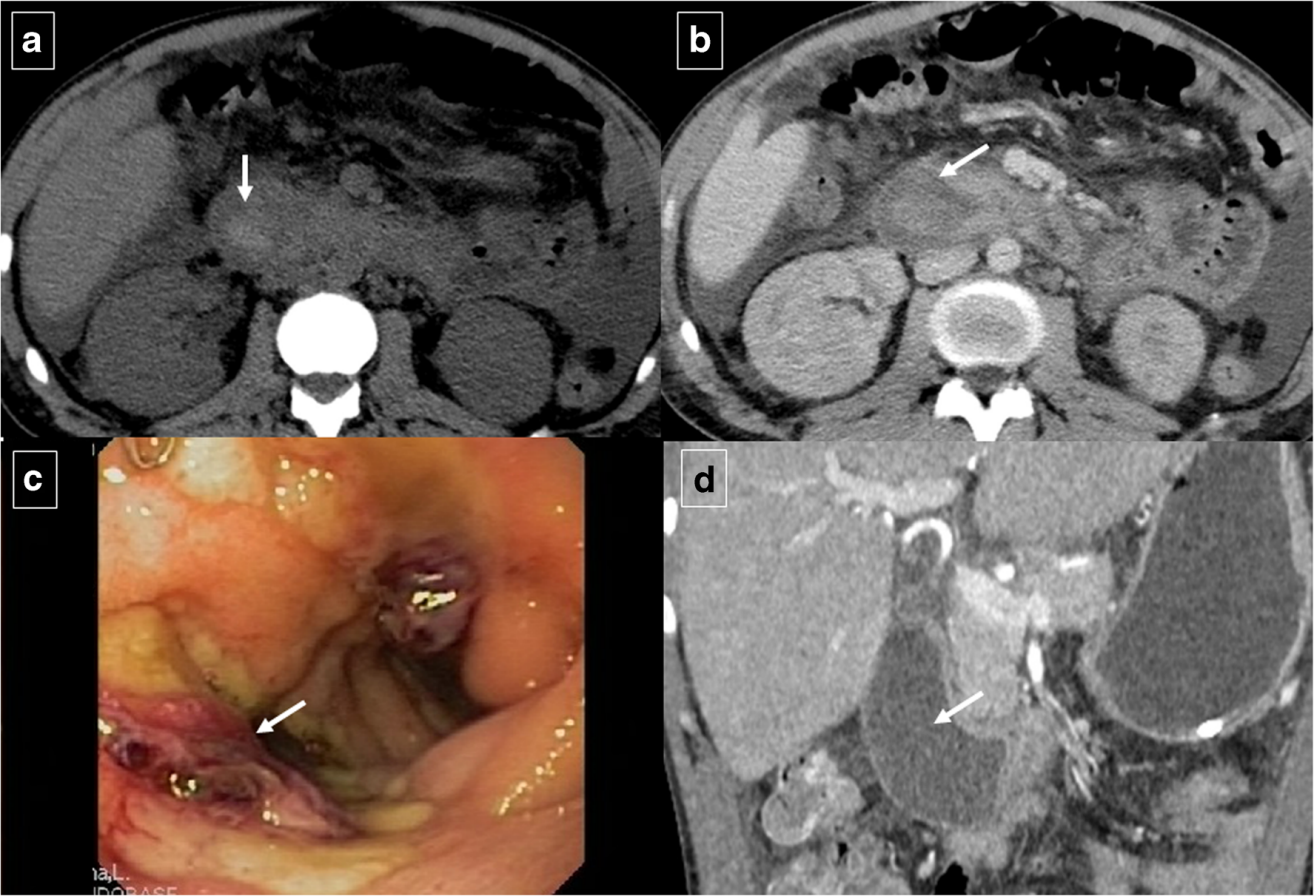
Intramural duodenal hematoma in a 28-year-old man with a history of cocaine abuse. **a** Unenhanced CT shows a spontaneous hyperdense thickening of the duodenal wall (arrow). **b** Axial contrast-enhanced CT shows a heterogeneous and intramural duodenal masslike appearance (arrow). **c** Detail of the endoscopic study in the initial diagnostic. **d** Reformatted coronal contrast-enhanced CT image of the same patient a few days before show a predominant hypodense expansive masslike appearance (arrow) causing a mass effect of the duodenal lumen with gastric dilation

Hematomas essentially affect the second and third part of the duodenum [7]. In addition to the mechanisms described above, hematomas can develop in relation to anticoagulant therapy, Henoch-Schonlein purpura, and blood dyscrasias. On CT and due to clot formation, intramural hematomas appear as a highly attenuated mass in the acute phase, but the breakdown of blood products leads to decreasing attenuation in the chronic phase (Fig. 22). This feature, with a lack of contrast-enhancement and the smooth and well-defined margins without mucosal ulcerations, differ isolated duodenal hematomas from real masses.

**Fig. 23:**
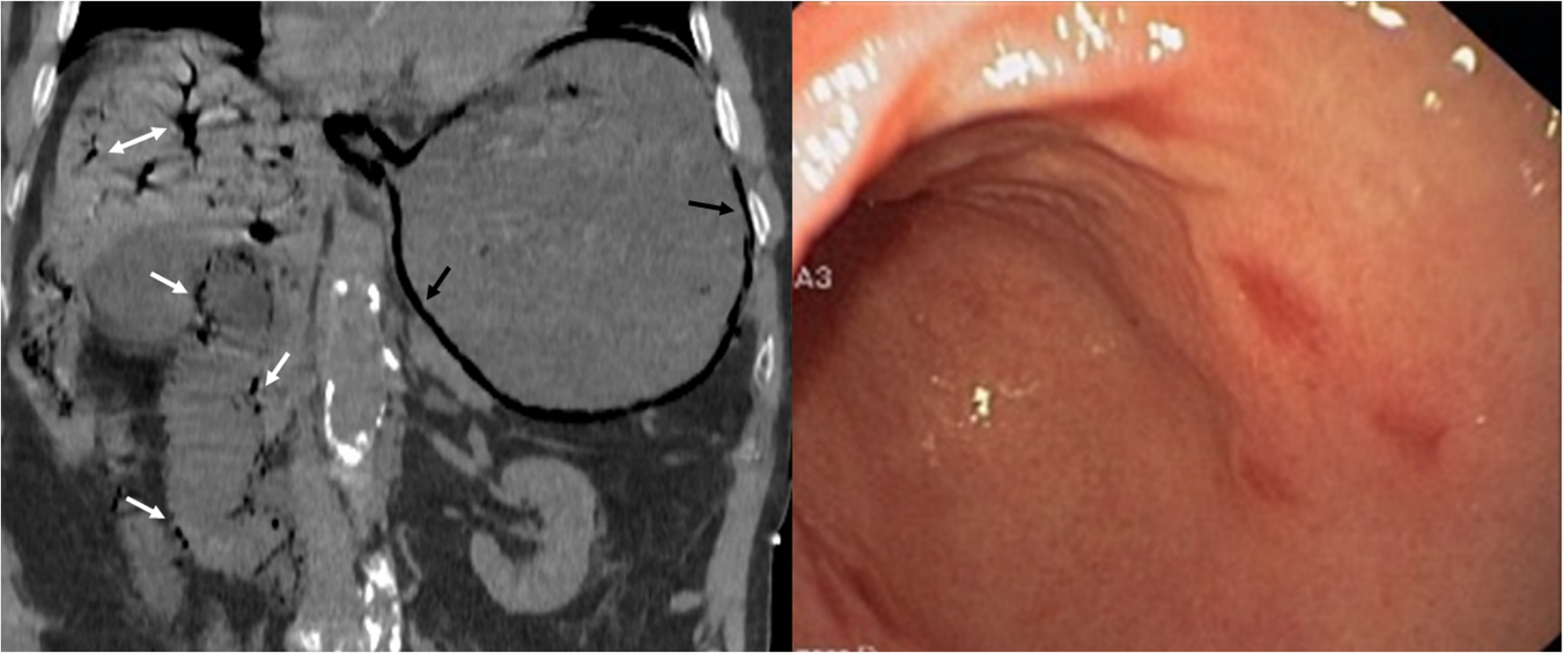
A 58-year-old man with coffee ground vomitus, hypotension, and abdominal pain. Reformatted coronal unenhanced CT image shows a gastroparesis caused by extensive intramural bowel gas (pneumatosis intestinalis) that affects the entire stomach (black arrows) and duodenum (white arrows). Also note gas in the portal vein (double arrow). Upper endoscopy shows a necrotic gastric and duodenal mucosa

Ischemic duodenitis, caused by splanchnic arterial insufficiency, is very rare because the duodenum has a rich collateral blood supply. Ischemic duodenitis is observed only when at least two of three main splanchnic arteries are occluded or severely stenosed with severe atheromatous disease [24]. At CT, one can see segmental thickening of the duodenal wall and a lack of mucosal enhancement; submucosal haemorrhage or hyperaemia can also be present. Asymmetric and discontinuous thickened duodenal wall and abnormalities of the folds are more typical of Crohn’s disease. Other typical findings are pneumatosis intestinalis and gas in the mesenteric or portal vein. (Fig. 23). Risk factors such as hypertension and coronary artery disease and a high clinical suspicion are key to early diagnosis.

**Fig. 24:**
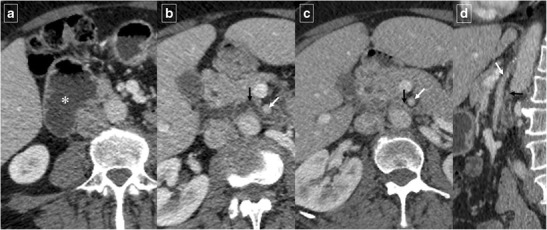
A 31-year-old woman with suspected superior mesenteric artery (SMA) syndrome. Axial contrast-enhanced CT images (a, b, and c) show a dilated proximal duodenum (*) with abrupt narrowing of the third portion (black arrows) corresponding with the course of SMA (white arrow). Reformatted sagittal contrast-enhanced CT image demonstrates compression of the third portion of the duodenum (black arrow) between the SMA (white arrow) and aorta

SMA syndrome is characterized by compression of the third portion of the duodenum. This uncommon entity (incidence 0.1%–0.3% [3]) is responsible for subacute or recurrent forms of obstruction secondary to an abnormally acute SMA angle. The fundamental underlying cause is acute loss of retroperitoneal fatty tissue. Diagnostic criteria on imaging include acute angulation of the SMA (aortomesenteric angle <22° and aortomesenteric distance <8–10 mm) with obstruction of third part of the duodenum (Fig. 24). Given the rarity of the syndrome, the CT findings must be interpreted in the context of the patient’s symptoms, and the absolute SMA angle may not be the best indicator of this entity [25].

## Conclusion

Imaging can play an important role in the multidisciplinary identification and management of duodenal diseases, determining the exact location and extent of the disease as well as confirming the presence of an actual expansive lesion when upper gastrointestinal endoscopy can only detect a mass effect in the duodenal lumen. Although the imaging features of some duodenal processes are nonspecific, awareness of the common sites of involvement and imaging presentation together with correlation with clinical presentation can often help in reaching the correct diagnosis.
